# Application of Modern Clinical Risk Scores in the Global Assessment of Risks Related to the Diagnosis and Treatment of Acute Coronary Syndromes in Everyday Medical Practice

**DOI:** 10.3390/ijerph18179103

**Published:** 2021-08-28

**Authors:** Lukasz Gawinski, Per Engelseth, Remigiusz Kozlowski

**Affiliations:** 1 Department of Management and Logistics in Health Care, Medical University of Lodz, 90-237 Lodz, Poland; 2 Narvik Campus, Tromsø School of Business and Economics, University of Tromsø, 8505 Narvik, Norway; pen008@uit.no; 3 Center of Security Technologies in Logistics, Faculty of Management, University of Lodz, 90-237 Lodz, Poland; remigiusz.kozlowski@wz.uni.lodz.pl

**Keywords:** risk assessment system, myocardial infarction, treatment decision-making

## Abstract

This article presents an overview of contemporary risk assessment systems used in patients with myocardial infarction. The full range of risk scales, both recommended by the European Society of Cardiology and others published in recent years, is presented. Scales for assessing the risk of ischemia/death as well as for assessing the risk of bleeding are presented. A separate section is devoted to systems assessing the integrated risk associated with both ischemia and bleeding. In the first part of the work, each of the risk scales is described in detail, including the clinical trials/registers on the basis of which they were created, the statistical methods used to develop them, as well as the specification of their individual parameters. The next chapter presents the practical application of a given scale in the patient risk assessment process, the timing of its application on the timeline of myocardial infarction, as well as a critical assessment of its potential advantages and limitations. The last part of the work is devoted to the presentation of potential directions for the development of risk assessment systems in the future.

## 1. Introduction

The 21st century is a time of tremendous development of medicine, and a period of implementing newer pharmacological and technological solutions into treatment. Miniaturization and advances in electronics and technology have allowed the use of interventional treatment methods in modern medicine on an unprecedented scale. Cardiology has been a leader in this field for many years. There has been a huge evolution in the treatment of heart disease over the past decades. The best example of this is myocardial infarction (MI). The process of treating this disease has gone from the use of simple drugs and the obligatory several-day bed regimen, through targeted fibrinolytic drugs, to modern, highly technologically developed methods of interventional cardiology allowing the patient to leave the hospital after a few days [1]. The simultaneous rapid development of cardiology and technology resulted in the creation of special logistic systems creating a network of medical units focused on optimizing the treatment process of patients with MI [2]. All the above-mentioned achievements make the modern methods and results of treating patients with MI radically different from those used and achieved at the beginning of the 21st century, which directly translated into a decrease in mortality rates due to MI over the last decades [3,4]. Unfortunately, despite the undoubted benefits of using modern methods of treatment, the other side of the coin should also be seen. The development of medical technology, the implementation of new devices and an increasingly aggressive approach to the treatment of MI is inevitably associated with the appearance of a completely new type of adverse events and complications [5]. Of course, in the final analysis of the population, the sum of benefits exceeds the potential threats; however, analyzing individual cases, complications, and adverse events may be the reason not only for prolonged hospitalization, but also for the patient’s death. Another aspect that is noteworthy is the suddenness of events and complete unpredictability in the treatment of MI. A clear distinction should be made between the risk of elective procedures (as part of the treatment of stable coronary artery disease) and the risk of procedures performed in terms of MI. The latter are initially burdened with a much higher risk of death and adverse events resulting from the severe clinical condition of the patient, as well as from aspects not related to the patient’s condition (time of the day when procedures are performed, experience of the staff on duty, staff fatigue). In some cases, the use of interventional cardiology methods, despite the indisputably proven indications and benefits, paradoxically may result in serious complications and actually shorten the patient’s life.

Currently, there is a lack of reliable tools that could be used by a physician, sometimes making a very difficult decision to apply or withdraw from interventional treatment [6]. The creation of a simple tool enabling a realistic assessment of the risk of complications and death of the patient would be very helpful for the physician during the clinical evaluation of the patient. The above facts were also noticed by the initiators of the guidelines of the European Society of Cardiology (ESC) regarding the diagnosis and treatment of both ST-elevation myocardial infarction (STEMI) [7] and without persistent ST segment elevation (NSTEMI) [8]. In the above documents, increasing importance is attached to the assessment of the risk of death of the patient, both during hospitalization and in the period after discharge from the hospital.

The body of a patient suffering from a heart attack treated with modern pharmacotherapy is a field of struggle between two opposing forces. On the one hand, it is a state of local excessive coagulation in the coronary artery associated with the release of pro-inflammatory and pro-thrombotic factors from the ruptured plaque, causing the formation of a blood clot blocking the flow in the coronary artery [9]. On the other hand, there is an opposing force associated with the pharmacological treatment used. The final result and effectiveness of treatment depends on the balance of the above factors. Of course, nowadays treatment of MI is not only pharmacotherapy, but also interventional cardiology methods, allowing for the implantation of a stent into the coronary artery, as well as mechanical removal of the thrombus from the artery [10]. By analogy with the two competing forces described above, it is possible to distinguish two main threats to the life of a patient with MI: myocardial ischemic events (primary on admission or recurrent during hospitalization) and massive bleeding. Modern risk assessment systems for patients with MI were constructed on a similar basis.

The aim of this study was present contemporary models of risk assessments in patients treated for MI, followed by an attempt to critically analyze the possibility of using these risk scales in everyday medical practice, pointing to the benefits and gaps, as well as showing the directions of development for new ones.

## 2. Material and Methods

This paper is a systematic review of literature on the applied clinical scores related to both the risk of an ischemic event/death and bleeding during the treatment of all forms of acute coronary syndrome (ACS). First, the scores recommended by the ESC in the guidelines for the treatment of MI will be discussed. Second, we will present the latest systems for assessing the risk of death or major adverse cardiovascular events (MACE), which are not recommended by ESC and based on large multicenter clinical trials conducted exclusively in the last decade. Data on the scales recommended by ESC were obtained on the basis of the recent STEMI/NSTEMI treatment guidelines [7,8]. In order to find other risk scores, PubMed (https://pubmed.gov, accessed on 26 August 2021) was used to search English language risk models that were developed in last decade and using the date patients with MI. This paper does not describe risk assessment models based on small, single-center studies or risk scales used in the assessment of patients with stable coronary artery disease (CAD) undergoing invasive procedures.

## 3. Results

The first attempts to create a model for assessing the risk of death in patients treated for MI began in the 1960s [11,12,13]. These models were developed at a time when reperfusion therapy was not yet used. Subsequent scores developed with the introduction of widespread use of fibrinolytic drugs [14,15,16,17]. Some of these scores were in the form of simple risk stratifications schemes that could be applied directly at the patient’s bedside without the use of a computer [14,15]. These systems were developed by using general measures of severity of illness, such as the Acute Physiology and Chronic Health [16], whereas others were based on expert opinion and prior investigation [14]. The 21st century is the era of medicine based on the principles of EBM and comprehensive guidelines indicating methods of diagnosis and treatment of particular diseases. Contemporary clinical scores applicable to risk assessment in a patient with ASC are presented in the ESC guidelines for the treatment of STEMI [7], NSTEMI [8], and guidelines for revascularization [18]. The recently available 2017 STEMI treatment guidelines raise the importance of introducing scores to assess the risk of death or recurrence of ischemic events in everyday clinical practice. The suggested scale is the TIMI score and the GRACE score. However, the guidelines do not give these suggestions an official class or recommendation.

### 3.1. Use of the TIMI Scale in the Risk Assessment of Patients with STEMI

The TIMI (thrombolysis in myocardial infarction) score is a clinical score developed 21 years ago to assess the short-term risk of patients with STEMI at hospital presentation [19]. The score assesses the risk of 30-day mortality after a heart attack. The TIMI risk score is simple and quick to use, convenient in everyday practice, and possible to use at the patient’s bedside. It does not require the use of computer devices or internet access. TIMI score was based on subsequent analysis of Intravenous nPA for Treatment of Infarcting Myocardium Early II (in TIME II) trial [20] which was multicenter, worldwide trial which enrolled patients with STEMI within 6 h of symptoms onset. Patients meeting the inclusion criteria were randomly assigned to fibrinolytic therapy with either lanoteplase or alteplase. Vital signs were assessed through 30 days and every 6 months. Using the multivariate regression analysis method, 16 independent predictors of mortality were identified. The discriminatory capacity of the full 16 variable regression model was assessed by using the area under the receiver operating characteristic curve (ROC) (c statistic) as an index of model performance (c statistics of 1 indicating perfect discrimination) [21]. The above described multivariate analysis model demonstrated a strong discriminatory capacity (c statistics 0.784). Ten variables, accounting for 97% of the predictive capacity of the multivariate model, constituted TIMI risk score. TIMI score is a simple arithmetic sum of points values assigned to independent risk factors:

1. age 64–75/ ≥75—2/3 points respectively,
2. systolic blood pressure (SBP) < 100 mmHg—3 points,
3. heart rate > 100 per min—2 points,
4. Killip class II-IV—2 points,
5. STEMI of anterior wall of heart or left bundle branch block (LBBB) in electrocardiography (ECG) recording—1 point,
6. diabetes or history of hypertension or angina—1 point,
7. weight of patient < 67 kg—1 point,
8. time to treatment > 4 h—1 point.

The maximum score is 14 points. Each score on the scale was assigned to mortality rate at 30 days (%): 0—0.8%, 1—1.6%, 2—2.2%, 3—4.4%, 4—7.3%, 5—12.4%, 6—16.1%, 7—23.4%, 8—26.8%, and >8—35.9%, respectively. The prognostic performance of the TIMI risk score was compared with the above mentioned 16 variable analysis model (c statistic 0.779 vs. 0.784). TIMI risk score was validated in the external dataset patients of TIMI 9 A/B trial [22,23] and showed similar prognostic capacity (c statistic 0.746). The TIMI score presented prognostic capacity comparable to both mentioned above multivariable model from GUSTO-I [17] (c statistic 0.803) and the risk score from TIMI 2 [14] (c statistic 0.753) (both tested in the InTIME II data set).

### 3.2. Use of the TIMI Risk Score in the Risk Assessment of Patients with NSTEMI

The paper introducing the clinical application of the TIMI score in the short-term risk assessment of patients with NSTEMI was published in 2000 [24]. The methodology, statistical analysis, and the way of creating the score were analogous to the TIMI score described above, used to assess the risk of patients with STEMI. The score was based on the analysis of two phase 3, international, randomized, double blind trials: the Thrombolysis in Myocardial Infarction (TIMI) 11B trial [25] and the Efficacy and Safety of Subcutaneous Enoxaparin in Unstable Angina and Non-Q-Wave MI trial (ESSENCE) [26]. There were four study cohorts: one test cohort and three validation cohorts.

A total of 1957 patients with NSTEMI/UA (unstable angina) were assigned to receive unfractionated heparin (test cohort) and 1953 to receive enoxaparin in TIMI 11B; 1564 and 1607 were assigned respectively in ESSENCE. The three validation cohorts were the unfractionated heparin group from ESSENCE and both enoxaparin groups. In both studies, for the purpose of creating the TIMI score, the following composite endpoint was adopted: all-cause mortality, new or recurrent MI, or severe recurrent ischemia prompting urgent revascularization. Endpoint incidence was assessed in the study cohort population within 14 days of randomization. Initially, 12 variables were selected as predictors of endpoint occurrence. In second stage, each factor was tested independently in a univariate logistic regression model; those that achieved a significance level of *p* < 0.20 were selected for testing in a multivariate stepwise logistic regression model.

The final TIMI score consists of seven factors: 65 years or older, at least three risk factors for coronary artery disease, prior coronary stenosis of 50% or more, ST segment deviation on ECG at presentation, at least two anginal events in prior 24 h, use of aspirin in prior 7 days, and elevated serum cardiac markers. The TIMI risk score is an arithmetic sum of points values assigned to particular risk factors where a value of 1 is given when a factor is present and 0 when it is absent. For the point on coronary stenosis, a value of 0 was assigned if no cardiac catheterization had been previously performed or if a prior cardiac catheterization revealed no coronary stenoses of 50% or more; a value of 1 was assigned if a prior cardiac catheterization revealed at least 1 coronary stenosis of 50% or more. The frequency of the assumed composite endpoint increased significantly as the TIMI risk score increased in the test cohort in TIMI 11B: 4.7% for a score of 0/1; 8.3% for 2; 13.2% for 3; 19.9% for 4; 26.2% for 5; and 40.9% for 6/7. The C statistic for the model in the test cohort was 0.65. The pattern of increasing event rates with increasing TIMI risk score was confirmed in all three validation groups.

The use of the TIMI scale in the risk assessment of patients with NSTEMI is currently not recommended. The 2015 NSTEMI guidelines for infarction treatment also mention the TIMI score, however the latest 2020 guidelines [8] explicitly recommend other scales to assess the risk of these patients.

### 3.3. Use of the GRACE Risk Score in Assessing the Risk of Patients with MI

Another scale recommended by the ESC for risk assessment in patients with MI is the GRACE risk score. This score applies to both STEMI and NSTEMI patients. Currently, due to the very good discriminative performance value, the GRACE score is the basic score recommended in the assessment of a patient with NSTEMI. In the ESC guidelines, it received a class IIa, level of evidence (LOE): B recommendation. In its assumption, it assesses the risk of myocardial ischemia and is based on the GRACE registry analysis [27]. GRACE (the Global Registry of Acute Coronary Events) is a large, prospective, multinational observational study of patients hospitalized with ACS. A total of 18 cluster sites in 14 countries in North America, South America, Europe, Australia, and New Zealand were collaborating in GRACE. Patients were followed up at 6 months after hospital discharge to identify recurrent coronary events, use of various medications, and mortality. Importantly, this registry included unselected patients with all forms of MI, which made it a very good reflection of the general population. Over time, several successive versions of GRACE scores have been developed to assess both short-term and long-term risk of death in patients with MI. Initially, these scores were used as a printed nomogram, and later internet calculators and mobile applications were developed. In fact, all subsequent models of the GRACE score are based on the same or a similar group of risk factors assessed on admission to the hospital; however, the weight of these variables differs depending on the version of the score. The original version of the GRACE risk score was used to assess the in-hospital risk of death in a patient with MI [28]. The score was based on the analysis of data from the GRACE registry (11389 patients with MI, both STEMI and NSTEMI, enrolled in the study in the period from 1 April 1999 to 31 March 2001). The score was created based on a model of a linear relationship between a given predictor and the risk of death. These relationships were assessed using the methods of multivariate logistic regression analysis. Finally, eight variables accounting for 89.9% of the prognostic information were identified: four continuous variables: age, SBP, heart rate, serum creatinine; three binary variables: cardiac arrest on admission, increased concentrations of cardiac biomarkers, and ST-segment deviations on admission; and one categorical variable: Killip class on admission.

Each risk factor has an appropriate number of points, and the values of continuous variables are assigned to a specific range of values and a corresponding number of points is assigned. The sum of the individual values of the risk factors allows us to read the risk of in-hospital death as a percentage. For example: the sum of points below 60 is related to the risk of ≤0.2%, the value of 140 points—2.9%, 200 points—18%, ≥250 points—risk ≥52%. The c statistic of this model is 0.84, indicating excellent discrimination. This model performed well in all major subgroups. The c statistics for patients with STEMI was 0.83 in comparison to NSTEMI patients (0.82), for patients with (0.81) and without (0.83) elevated cardiac markers at presentations, for patients 65 years or younger (0.78) in comparison to patient older than 65 years (0.82). External validation of the model was performed on a subsequent sample of 3972 patients from GRACE registry (c statistic 0.85) and on a data set from the GUSTO IIb study (c statistic 0.79), which confirmed excellent discrimination. This scale was presented in the form of a printed nomogram, which allows for quick calculation of the risk of in-hospital death in patients with MI.

The next version of the GRACE score allows for the assessment of the patient’s risk of death within 6 months of hospital discharge [29]. This scale, like the previous one, was based on the analysis of data from the GRACE registry (15,007 patients MI, both STEMI and NSTEMI, enrolled in the study in the period from 1 April 1999 to 31 March 2002). As the endpoint of the study, the incidence of all-cause deaths within 6 months of hospital discharge following an MI was assessed. The model was created by using a multivariable Cox proportional hazards regression backward elimination technique. The model was validated on the consecutive 7638 patients enrolled in GRACE between 1 April 2002 and 31 December 2003. Statistic c for the study cohort and validate cohort was 0.81 and 0.75 (respectively), which confirmed the good discriminating value of the scale. C statistic for different types of MI (STEMI, NSTEMI, UA) were also similar. The finally developed risk prediction tool for all forms of ACS included nine variables: age, history of MI, history of congestive heart failure (CHF), pulse rate, SBP, serum creatinine concentration, cardiac biomarkers concentration, ST segment depression, and not having PCI performer in hospital. The continuous variables were grouped into ranges, each range of continuous variables and each binary variable were assigned an appropriate number of points. The score value was calculated by summing up the values of individual variables. The attached reference plot nomogram shows the risk of death corresponding to the total risk score. It should be noted that this scale can be used only when the patient is discharged from the hospital (only then we can assess the occurrence of all risk factors), and to calculate the point value of the score for continuous variables, we take into account the parameters or results of laboratory tests on admission to the hospital (first laboratory tests performed).

The next version of the GRACE scale was created only in electronic form (a calculator on the website, an application for a mobile phone) [30]. It is identified as GRACE 1.0. The scale is a combination and extension of the existing models of the GRACE scale, because it allows for the calculation of the risk of death and death or non-fatal MI, either

1. in the hospital period,
2. in the period from admission to 6 months after discharge,
3. in the period from hospital discharge to the 6th month of follow-up.

In total, it makes it possible to calculate six different risk values. This scale, like the previous one, was based on the analysis of data from the GRACE registry (21,688 patients with MI, both STEMI and NSTEMI, enrolled in the study in the period from April 1999 to September 2005). The study adopted two main endpoints: all cause death or the composite measure of death or non-fatal MI during admission to hospital or after discharge. A Cox regression model was used to calculate hazard ratios and 95% confidence intervals to examine the individual relations between a particular predictor and death and death or MI during follow up. The final model for the risk score of death or death and death or MI was constructed using a multiple Cox regression backward analysis. Statistic c for the final model of score was 0.70 (for the composite end point: death and death or MI from hospital admission to 6 month follow up) and 0.82 (for end point: all cause of death from hospital admission to 6 month follow up). The model was subjected to internal validation on the consecutive 22,122 patients enrolled in GRACE cohort, as well as external validation using GUSTO IIb data set of 12,142 patients with ACS, confirming the very good discriminating value of the scale. C statistic for internal validation was 0.81 and 0.73 (all cause of death and death or MI from hospital admission to 6 month follow up, respectively). 

Finally, the prepared risk calculator (in electronic or online form) includes individual risk factors:

1. for the risk of death and death or MI in the in-hospital period/in the period from admission to the sixth month of observation: age, heart rate, SBP, serum creatinine concentration, Killip class, ST segment deviation, cardiac arrest at admission, elevated cardiac enzymes/markers;
2. for the risk of death and death or MI in the period from discharge to 6 months of follow-up: age, heart rate, systolic blood pressure, serum creatinine concentration, CHF, in-hospital primary coronary intervention (PCI), in-hospital coronary artery bypass grafting (CABG), past history of MI, ST segment depression, elevated cardiac enzymes/markers.

Another version of the GRACE scale, the last in the series and published in 2014, is version 2.0 [31]. This scale allows for risk assessment of a total of five different risk values: typical for the previous GRACE scale values—in-hospital death risk, risk of death within 6 months of admission—and new possibilities of risk values—risk of death within 1 year after admission to hospital, risk of death or recurrent MI within one year of MI, risk of death within 3 years of admission to hospital. The GRACE 2.0 scale, like its predecessor, is only available in the form of an electronic or online risk calculator. The scale was based on the analysis of data from the GRACE registry (32,037 patients with MI, both STEMI and NSTEMI, enrolled in the study from January 2002 to December 2007; additionally, for the purpose of analyzing the risk of death within 3 years from admission to the hospital, a separate group of 1274 patients was analyzed). The final model included eight classic variables for the previous versions of the GRACE scale: age, heart rate, SBP, serum creatinine concentration, Killip class, ST segment deviation, cardiac arrest at admission, elevated troponin or other necrosis cardiac biomarkers. The novelty of the GRACE 2.0 risk score is the possibility of an alternative replacement of the serum creatinine concentration value (in the absence of data) with the information about renal failure in the patient (in binary mode: 0/1) and the Killip class with the information about the patient’s previous use of diuretics (in the binary: 0/1). The above solution was introduced earlier in the mini GRACE scale. The above-mentioned scale is based on the analysis of 64,312 patients from the MINAP database (Myocardial Ischaemia National Audit Project). It has been proven that this approach also demonstrated good performance (c statistic 0.825) [32]. Another novelty of the GRACE 2.0 scale is the possibility of assessing the risk in a longer time range, namely after 1 and 3 years after a heart attack. For the first time in the history of the development of the GRACE scale, it was possible to assess the risk of death in the long term. Another novelty is the introduction to the analysis of non-linear methods of assessing the relationship between risk and a given predictor, which significantly improved the discriminating value of the obtained model. The value of the c statistics for the GRACE 2.0 scale—a model for assessing the risk of death within 1 year of admission to hospital, a model for assessing the risk of death or MI within 1 year of admission, and a model for assessing the risk of death within 3 years of admission to hospital was: 0.82, 0.74, and 0.78 (respectively). The scale was externally validated on a population of 3059 patients from the FAST—MI registry (French Registry of Acute ST—elevation and non-ST-elevation Myocardial Infarction), confirming the good performance.

### 3.4. Other Risk Scores Assessing the Risk of Ischemia/Death in Patients with MI (Not Included in the ESC Guidelines)

Over the last 20 years, many risk scores have been developed to assess the risk of death in patients with MI. Some of them are completely inapplicable nowadays due to the dynamic development of methods of treating MI. The beginning of the 21st century is a period of a breakthrough in cardiology, the end of the thrombolytic treatment era and the rapid development of interventional cardiology methods. Risk assessment systems that were created at the beginning of the century (2000–2010) do not reflect the current reality, both in terms of the methods of treatment used, the scale of implementation of invasive treatment, pharmacotherapy (new antiplatelet and anticoagulant drugs), population characteristics, as well as technological development in the field of devices and stents used in everyday practice of the catheterization laboratory. A new generation of drug eluting stents (DES) was introduced for treatment, and the use of bare metal stents (BMS) has practically ceased. Some scales, such as the GRACE risk score described above, were systematically updated, which allowed them to remain credible today. At the beginning of the 21st century the following risk assessment systems were created: PAMI [33], SIMPLE risk index [34], CADILLAC [35], ZWOLLE [36], RISK-PCI [37]. For several years since their creation, these scales have been described in numerous and detailed publications, their mutual comparisons and comparisons with the GRACE scale have also been made [38,39]. In this paper, we will present the latest systems for assessing the risk of death or major adverse cardiovascular events (MACE), which are based on large multicenter clinical trials conducted in the last decade.

#### 3.4.1. TIMI DYNAMIC Risk Score

The TIMI DYNAMIC risk score [40] was published in 2013 and is a kind of extension and supplementation of the original TIMI scale [19]. It was supposed to be a simple, bedside clinical scale allowing to assess the risk of death in a patient with STEMI within 1 year of discharge. The TIMI DYNAMIC scale is a prospectively validated scale for the reclassification of patients with STEMI based on in-hospital events. It consists of the classic risk factors for the TIMI scale, which are based on the parameters collected on admission to the hospital, and new variables which were major clinical events occurring during the hospitalization (in hospital events). The scale is based on the analysis of data from the ExTRACT-TIMI 25 study (Enoxaparin and Thrombolysis Reperfusion for Acute Myocardial Infarction Treatment)—a double-blind, international study which randomly assigning 20,506 patients with STEMI to either enoxaparin or unfractionated heparin as an adjunctive therapy to fibrinolysis [41]. The study endpoint was death or recurrence of MI within one year of discharge from the hospital. Each new variable (in hospital events) was first subjected to univariate Cox analysis (to confirm significance) and then tested in the multivariate Cox analysis to assess the effect on the risk of death over a 1-year follow-up period. Ultimately, the TIMI DYNAMIC scale included six new variables. Baseline variables taken from original TIMI scale were retested to reconfirm the significance. Each variable was assigned an integer value based on the odds ratio, and the final score was the sum of these values. The TIMI DYNAMIC scale consisted of eight variables assessed on admission to hospital: age (65–74 years—2 points, >75 years—3 points), SBP (<100 mmHg—3 points), heart rate (>100 bpm—2 points), Killip class II-IV—2 points, STEMI of anterior wall of heart or LBBB in ECG recording—1 point, diabetes or history of hypertension or angina—1 point, weight of patient <67 kg—1 point, time to treatment >4 h—1 point, and another six variables assessed at discharge: recurrent MI—1 point, stroke—5 points, major bleed—1 point, arrythmia (atrial fibrillation, ventricular fibrillation, ventricular tachycardia)—2 points, renal failure—3 points, CHF or cardiogenic shock—3 points. The maximum number of points to be obtained is 29. The absolute risk of death during 1 year from discharge in relation to the TIMI DYNAMIC score was assessed as below: 0–1 point—1.3%; 2 points—2.3%; 3 points—3.6%; 4 points—5.5%, 5 points—7.8%; 6–7 points—13.5%; ≥8 points—24.8%. The value of the c statistic for the TIMI DYNAMIC scale is 0.76, which confirms a good discriminating value of this scale. The scale was externally validated on a cohort of 3 454 patients with STEMI infarction included in the TRITON TIMI 38 study [42]. The predictive capacity of TIMI Dynamic risk score remained consistent for 1-year mortality with c statistic of 0.81.

#### 3.4.2. ACTION Registry-GWTG Risk Model

Another contemporary risk assessment system is the ACTION Registry-GWTG risk model [43]. The “Acute Coronary Treatment and Intervention Outcomes Network (ACTION) Registry-Get With the Guidelines (GWTG)” (AR-G) score was constructed using data of both STEMI and non-STEMI patients to predict in-hospital mortality. The scale is based on the analysis of 254,066 patients from 655 hospitals included in the ACTION-GWTG registry in the period from January 2012 to December 2013. ACTION Registry-GWTG is a voluntary registry kept in the United States receives data on patients admitted to hospital with a diagnosis of MI (both STEMI and NSTEMI) [44]. Study population was divided into a research cohort (60%) and a validation cohort (40%). The model was created using hierarchical logistic regression from the selected variables. Ultimately, the scale included nine variables with points assigned for each value for each parameter: age (<40 years —0 points, 40–49—3 points, 50–59—7 points, 60–69—10 points, 70–79—13 points, 80–89—17 points, ≥90 years—20 points); SBP (>200 mmHg—0 points, 181–200—3 points, 171–180—5 points, 161–170—7 points, 151–160—9 points, 131–150—11 points, 121–130—13 points, 111–120—15 points, 91–110—16 points, ≤90—19 points); creatinine clearance (CrCl) (≥90—0 points, 60–<90—4 points, 45–<60—8 points, 30–< 45—11 points, <30 or dialysis—15 points); cardiac arrest (yes—14 points); cardiogenic shock (yes—13 points); heart rate (≤40 bpm—0 points, 41–60—1 points, 61–70—2 points, 71–80—3 points, 81–100—4 points, 101–110—5 points, 111–130—7 points, 131–150—8 points, >150—9 points); heart failure (yes—5 points); STEMI (yes—5 points); troponin ratio (<10—0 points, 10–< 20—1 points, 20–<30—2 points, ≥30—3 points). Troponin ratio was defined as the baseline troponin value divided by the local laboratory-specific upper limit of normal. The sum of the individual values gave a total score. The mortality rates in patients with risk scores <30, 30–39, 40–49, 50–59, and >59 were 0.4%, 1.7%, 5.5%, 18.5%, and 49.5%, respectively. The scale model was only subject to internal validation. The value of the c statistic for both derivation cohort and validation cohort is 0.83, which confirms a good discriminating value of this scale.

#### 3.4.3. EPICOR Risk Score

EPICOR web-based risk calculator [45] is used to assess the risk of death within 2 years of hospital discharge after MI and is a continuation and development of the previous version of this system for assessing the risk of death within 1 year of MI [46]. The scale was developed based on the analysis of the EPICOR study (long-term follow up of antithrombotic management patterns in acute coronary syndrome patients) [47] and EPICOR Asia [48]. Both of these studies are prospective, international, observational studies which enrolled 23,489 patients from 28 countries across Europe, Latin America, and Asia, who were hospitalized for MI (STEMI, NSTEMI, and UA) within either 24 h (EPICOR) or 48 h (EPICOR Asia) of symptom onset, and who survived to hospital discharge. The risk score model was created using identified predictive variables and forward stepwise Cox regression. The model was internally validated using a bootstrap method. In the original model, 17 independent mortality predictors were determined: age, low ejection fraction (EF), no coronary revascularization/thrombolysis, elevated serum creatinine concentration, poor EQ-5D score, low hemoglobin, previous cardiac or chronic obstructive pulmonary disease, elevated blood glucose, on diuretics or an aldosterone inhibitor at discharge, male sex, low educational level, in-hospital cardiac complications, low body mass index (BMI), STEMI diagnosis, and Killip class. EuroQoL (EQ-5D) is a quality of life generic questionnaire which grades each of five parameters: mobility, self-care, ability to perform usual activities, pain/discomfort, and anxiety/depression as ‘no problem’ (zero points), ‘moderate’ (one point) or ‘a severe limitation’ (two points). A risk score is calculated from the risk coefficients of the linear predictors for the overall model. A simplified model was also created to facilitate the practical application of the scale, which contained only 11 variables (six of the variables removed which had a somewhat lesser impact on patient risk: on diuretics and on aldosterone inhibitor at discharge, education level, in-hospital complications, BMI and Killip class. The EPICOR risk-scoring system provided excellent discrimination capacity (c statistic 0.80, 95% CI (0.79–0.82)). A simplified risk model with 11 predictors gave only slightly weaker discrimination (c statistic 0.79, 95% CI (0.78–0.81)).

#### 3.4.4. ACEF Risk Score

Originally the ACEF scale (age, creatinine, and EF) was designed to assess the risk of elective cardiac surgery [49]. Due to its simplicity and the possibility of quick application at the patient’s bedside, a number of attempts have been made to adapt this scale to the price of the risk associated with other groups of patients and other clinical situations: patients undergoing stent implantation [50], and particularly in challenging patient subgroups such as those with left main disease [51]; bifurcation lesions [52]; heavily calcified lesions undergoing rotational atherectomy [53]; chronic total occlusions [54]; and patients with severe aortic stenosis undergoing transcatheter aortic valve replacement [55]. The scale value is calculated using the following formula: age/left ventricular ejection fraction (LVEF) + 1 (if creatinine >176 μmol/L). The use of the ACEF scale in the assessment of patients with MI was confirmed on the basis of the prospective, multicenter Swiss ACS cohort, which consecutively enrolled (between 12.2009 and 10.2012 at four university hospitals in Switzerland) 2168 patients undergoing coronary angiography for ACS (STEMI or NSTEMI/UA) [56]. Coronary revascularization by either PCI or CABG was performed according to current guidelines and recommendations. The primary endpoint of the study was all-cause mortality. The secondary endpoint was major adverse cardiac and cerebrovascular events (MACCE) defined as a composite of all-cause mortality, nonfatal MI, clinically indicated repeat coronary revascularization, definitive stent thrombosis, and transient ischemic attack/stroke [57]. Optimal ACEF score cut-off values were calculated by decision tree analysis, and patients were divided into low-risk (≤1.45), intermediate-risk (>1.45 and ≤2.0), and high-risk groups (>2.0). Thus, the score result does not indicate the absolute risk of death, but only classifies the patient into one out of three groups with increasing risk. Multivariate Cox proportional hazards regression models were calculated for 30-day and 1-year rates of mortality and MACCE both for continuous ACEF score values and ACEF score groups. The bootstrap re-sampling technique was used for internal validation. Cumulative incidence rates of 1-year mortality and MACCE according to ACEF score groups were estimated with the Kaplan-Meier method. In multivariate analysis, the ACEF score emerged as an independent predictor of 30-day rates of all-cause mortality (adjusted HR 3.35, 95% CI 2.61–4.30, *p* ≤ 0.001), MACCE (adjusted HR 2.30, 95% CI 1.85–2.87, *p* ≤ 0.001), and transient ischemic attack/stroke (adjusted HR 1.96, 95% CI 1.07–3.60, *p* = 0.03), as well as an independent predictor of 1 year rates of all-cause mortality (adjusted HR 3.53, 95% CI 2.90–4.31, *p* ≤ 0.001), MACCE (adjusted HR 2.23, 95% CI 1.88–2.65, *p* ≤ 0.001), and transient ischemic attack/stroke (adjusted HR 2.58, 95% CI 1.71–3.89, *p* ≤ 0.001). The analysis of the study also confirmed that the ACEF score achieved a similar predictive performance as the GRACE and CRUSADE scores. It was finally confirmed that the ACEF score independently predicts short- and long-term survival and adverse events in patients presenting with ACS referred for coronary revascularization.

### 3.5. Clinical Scales to Assess the Risk of Bleeding during the Treatment of MI

Undoubtedly, the use of dual antiplatelet therapy (DAPT) reduces the number of ischemic episodes in patients undergoing coronary stent implantation, but at the same time increases the risk of bleeding [58]. The latest ESC guidelines recommend a 12-month duration of DAPT in patients after both STEMI and (class I LOE: A). Bleeding is the most common complication after stent implantation and it shortens the life span, extends hospitalization, and lowers the quality of life [59]. Bleeding is also a common problem complicating treatment of MI. Clinical trials involving patients with NSTEMI demonstrate that major bleeding is associated with a 5-fold increase in 30-day mortality [60]. Assessing the risk of bleeding in an MI patient is therefore an important part of the treatment process and is a major challenge for the modern physician. The ESC guidelines for the treatment of NSTEMI [8] suggest the use of appropriate risk scores to assess the risk of bleeding (class IIb, LOE: B). The profile and characteristics of bleeding in a patient with MI change over time: in the in-hospital period there is a predominance of access site bleeding, related with the coronary intervention, and in the post-discharge period there is a predominance of gastrointestinal bleeding, related with antiplatelet therapy [61]. The risk factors for bleeding also change over time after implantation of stents. A similar division can also be seen in the risk scores of bleeding: scores assessing short-term risk (in-hospital, 30 days from admission to hospital) and scores assessing long-term risk (approximately 1 or 2 years after MI). The latter are most often scores integrating the assessment of risk related to ischemia and bleeding and are used to assess the optimal duration of DAPT.

#### 3.5.1. The CRUSADE Risk Score

The Crusade [62] score is a bleeding risk assessment system recommended by the ESC guidelines for the treatment of NSTEMI [8]. The scale is based on eight parameters (clinical data, laboratory test results, history data) and is intended to be used during the admission of a patient with MI to the hospital to assess the risk of major bleeding. The scale was based on the analysis of “can rapid risk stratification of unstable angina patients suppress adverse outcomes with early implementation of the ACC/AHA” guidelines (CRUSADE) Quality Improvement Initiative database of high-risk NSTEMI patients admitted to U.S. hospitals [63]. The analysis population consisted of 89,134 patients enrolled across 485 U.S. sites from February 2003 through December 2006. The study population was then divided randomly into a derivation cohort (80%, *n* = 71,277) and a validation cohort (20%, *n* = 17,857). The main aim of the study was to assess the relationship between individual covariates and the fact of major bleeding. Intracranial hemorrhage, documented retroperitoneal bleed, hematocrit (HCT) drop ≥12% (baseline to minimum value), any red blood cells (RBC) transfusion when baseline HCT ≥ 28%, or any RBC transfusion when baseline HCT < 28% with witnessed bleed was considered major bleeding in the clinical analysis. Potential variables with clinically and statistically significant univariate relationships with major bleeding were included in the multivariate model.

The CRUSADE bleeding score was developed by assigning a weighted integer to each independent predictor based on its coefficient in the final model. A point score for each patient is calculated by summing the weighted integers (range 1–100 points). Finally, the variables assessed on the CRUSADE scale included: baseline HCT (%) (<31—9 points, 31–33.9—7 points, 34–36.9—3 points, 37–39.9—2 points, ≥40—0 points); CrCl (mL/min) (≤15—39 points, >15–30—35 points, >30–60—28 points, >60–90—17 points, >90–120—7 points, >120—0 points); heart rate (bpm) (≤70—0 points, 71–80—1 points, 81–90—3 points, 91–100—6 points, 101–110—8 points, 111–120—10 points, ≥121—11 points); sex (male 0 points, female 8 points); signs of CHF at presentation (no—0 points, yes—7 points); prior vascular disease (no—0 points, yes—6 points); diabetes mellitus (no—0 points, yes—6 points); and SBP (mm Hg) (≤90—10 points, 91–100—8 points, 101–120—5 points, 121–180—1 points, 181–200—3 points, ≥201—5 points). CrCl (mL/min) was estimated using the Cockcroft-Gault equation. CHF was defined as signs of CHF at presentation indicated by exertional dyspnea, orthopnea, shortness of breath, labored breathing, fatigue at either rest or with exertion, rales >1/3 of the lung fields, elevated jugular venous pressure, S3 gallop, or pulmonary congestion on x-ray believed to represent cardiac dysfunction. Prior vascular disease was defined as either prior stroke or peripheral arterial disease. The bleeding score was also divided into quintiles: very low risk (≤20), low risk (21–30), moderate risk (31–40), high risk (41–50), and very high risk (>50). The individual quintiles were assigned the risk of major bleeding during hospitalization, assessed at 3.1% (very low risk), 5.5% (low risk), 8.6% (moderate risk), 11.9% (high risk), and 19.5% (very high risk).

It should be noted that the scale does not include information on treatment (conservative vs. invasive), information on the number of antithrombotic drugs taken (antiplatelet, anticoagulant, or glycoprotein IIb/IIIa inhibitors (GPI)) as well as the patient’s age. The CRUSADE bleeding score demonstrated a moderate discriminatory capacity in the derivation (c statistic 0.71) and validation cohorts (c statistic 0.70). Discriminative ability of the CRUSADE score was also confirmed in particular subgroups of patients: patients receiving ≥2 antithrombotic medications and those receiving <2 antithrombotic medications (c statistics 0.72 and 0.73, respectively) and patients receiving ≥2 antithrombotic medications treated with a conservative approach (no catheterization) versus treated with an invasive approach (catheterization) (c statistic 0.68 vs. 0.73). It is also worth noting that the intra-hospital mortality increases with the occurrence of major bleeding during hospitalization, as well as with the CRUSADE score.

#### 3.5.2. ACUITY Risk Score

The ACUITY risk score [64] is another of the scales recommended in the ESC guidelines for the treatment of NSTEMI. The scale assesses the risk of major bleeding within 30 days of admission to hospital. The scale is based on the analysis of databases from the ACUITY (acute catheterization and urgent intervention triage strategy) and HORIZONS-AMI (harmonizing outcomes with revascularization and stents in acute myocardial infarction) trials. ACUITY trial [65] enrolled 13,819 patients with moderate- and high-risk ACS (NSTEMI or UA) and was conducted to evaluate the safety of using various antithrombotic regimens before cardiac catheterization (heparin (unfractionated or enoxaparin) plus a GPI, bivalirudin plus a GPI, or bivalirudin monotherapy have been administered). All patients enrolled in the study underwent coronary angiography within 72 h of admission and were then qualified for PCI, CABG, or conservative treatment. HORIZONS-AMI trial [66] enrolled 3602 STEMI patients who presented within 12 h after symptom onset and were invasive treated. Patients were assigned to treatment with unfractionated heparin plus a GPI or to bivalirudin monotherapy. Antiplatelet therapy—aspirin and clopidogrel—was also used in both studies. Major bleeding was defined in both trials as intracranial or intraocular bleeding, access site hemorrhage requiring intervention, reduction in hemoglobin of ≥4 g/dL without or ≥3 g/dL with an overt bleeding source, reoperation for bleeding, or blood product transfusion. Bleeding was assessed as related or not related to CABG. The endpoint was major bleeding within 30 days and death within 1 year follow up. The integer risk score derived from multivariate logistic regression model consists of the summation of six integers from each baseline variable: gender (male 0 points, female 8 points); age (<50 years—0 points, 50–59—3 points, 60–69—6 points, 70–79—9 points, ≥80—12 points); serum creatinine concentration (mg/dL) (<1.0—0 points, 1.0–1.19—2 points, 1.2–1.39—3 points, 1.4–1.59—5 points, 1 6–1.79—6 points, 1.8–1.99—8 points, ≥2.0—10 points); white blood target count (giga/L) (<10—0 points, 10–11.9—2 points, 12–13.9—3 points, 14–15.9—5 points, 16–17.9—6 points, 18–19.9—8 points, ≥20—10 points); anemia (yes—6 points, no—0 points); clinical presentation (STEMI—6 points, NSTEMI—2 points, UA—0 points) representing the individual risk of bleeding if the patent received heparin plus a GPI. If bivalirudin is administered instead, 5 points are subtracted from the integer score. The four risk ranges of bleeding have been defined: low, moderate, high, and very high, corresponding to integer scores <10, 10–14, 15–19, and ≥20, respectively (with 30-day non-CABG-related bleeding rates of 1.9%, 3.3%, 6.9%, and 12.4%, respectively, in patients treated with a heparin plus a GPI and 0.7%, 2.0%, 3.7%, and 8.4%, respectively, in patients treated with bivalirudin monotherapy). C statistic of model was 0.74 and confirmed a good discriminative ability of this risk score. The relationship between the fact of bleeding and the risk of death within 1 year of follow-up was also analyzed. The model of nine independent predictors of 1-year mortality were identified using the multivariable Cox model (advanced age, elevated white blood cell count and serum creatinine concentration, diabetes mellitus (DM), reduced hemoglobin, smoking, sex, previous MI, and clinical presentation (STEMI, NSTEMI)). Antithrombotic treatment regimen was not an independent predictor of mortality. Both the occurrence of non-CABG-related major bleeding and MI within 30 days were independent predictors of subsequent mortality, when added to this multivariate model. The relationship between the severity of bleeding and the consequent risk of death has also been proven. It is worth noting that development of an isolated large hematoma ≥5 cm without more severe bleeding was not a statistically significant predictor of subsequent mortality. The relationship between CABG related major bleeding and subsequent mortality was analyzed separately, showing no statistically significant relationship between them (HR: 1.21, 95% CI: 0.81 to 1.80, *p* = 0.34).

#### 3.5.3. BleeMACS Risk Score

BleeMACS score is a simple clinical tool for bedside risk estimation of 1-year post-discharge serious bleeding in patients with MI [67]. A BleeMACS score calculator is available in a mobile app. The scale was based on the BleeMACS (bleeding complications in a multicenter registry of patients discharged with diagnosis of acute coronary syndrome) analysis [68]. BleeMACS was a retrospective, observational, multicenter study involving 15,401 consecutive patients from 15 hospitals (from 10 countries located in North and South America, Europe, and Asia) with ACS diagnosis and underwent in-hospital PCI, with data of follow-up during at least 1 year. Recruitment lasted from November 2003 through June 2014. The study population was divided into a derivation cohort (70% of patients) and an internal validation cohort (30% of patients). The primary endpoint of the study was serious spontaneous bleeding within the first year after hospital discharge. Serious spontaneous bleeding was defined as any intracranial bleeding or any other bleeding leading to hospitalization and/or RBC transfusion (≥1 unit). Bleeding and/or RBC transfusions related to procedures or surgeries were not considered as spontaneous bleeding. Potential predictors of bleeding risk were assessed by Fine-Gray proportional hazards regression analysis.

Finally, seven independent predictors of bleeding occurrence were identified and assigned the following weighted integer in the basis of its coefficient in the regression model: age (<67—0 points, 67–74.9—7 points, ≥75—9 points); arterial hypertension (7 points); previous history of bleeding (19 points); malignancy (8 points); vascular disease (6 points); admission hemoglobin (g/dL) (<11.0—18 points, 11.0–13.9—9 points, ≥14—0 points); and serum creatinine concentration (mg/dL) (<1.0—0 points, 1.0–1.49—3 points, ≥1.5—12 points). Definitions: hypertension: history of hypertension diagnosed and/or treated by a physician; vascular disease: prior stroke/transient ischemic attack and/or peripheral artery disease; history of bleeding: hospitalization due to a bleeding event prior to the qualifying ACS, and/or any serious bleeding occurring during hospitalization for the index ACS, defined as any TIMI major or TIMI minor bleeding event (TIMI bleeding classification [69]), any GUSTO moderate or severe bleeding event (GUSTO bleeding classification [70]), or any BARC type 3 bleeding event (BARC bleeding classification [71]); malignancy: any active cancer or any non-active cancer which was treated during the last 3 years.

A point score was calculated by summing the weighted integers (range 0 to 80 points). Patients were classified into quartiles of the BleeMACS risk score: very low-risk (≤7 points), low-risk (8 to 16 points), moderate-risk (17 to 24 points), and high-risk (≥25 points). The final value of cumulative incidence of bleeding during 1 year of observation can be obtained using an electronic calculator (application on a mobile device). The BleeMACS risk score has been thoroughly validated, both within the internal and external validation cohort. An external validation was performed using data from SWEDEHEART registry (Swedish Web-system for Enhancement and Development of Evidence-Based care in Heart Disease Evaluated According to Recommended Therapies) [72] which enrolled from 2003 to 2012 consecutive patients with the entire spectrum of ACS (UA, NSTEMI and STEMI) who underwent PCI (*n* = 96,239) and not (*n* = 93,150) during hospitalization. The final model of risk score exhibited good performance in the derivation (c statistic: 0.71, 95% CI 0.68–0.74) and internal validation cohorts (c statistic: 0.72, 95% CI 0.67–0.76), as well as in external validation cohort (the c statistic 0.65, 95% CI 0.64–0.66, for PCI treated patients; and 0.63, 95% CI 0.62–0.64, for non PCI treated patients).

#### 3.5.4. PARIS Risk Score

The PARIS scale is another risk assessment model that has not been included in the ESC guidelines. The purpose of the PARIS scale [73] was to create two separate models assessing both the risk of a bleeding and a thrombotic event within 2 years of PCI with DES implantation. The system of these 2 separate scales (more precisely, the balance between the scores achieved on both scales) can help the clinician decide on the duration of DAPT. A risk scale for major bleeding (MB) and risk score for coronary thrombotic events (CTE) was developed. The risk model was developed on the basis of the analysis of the patient population from the PARIS registry (Patterns of Non-Adherence to Anti-Platelet Regimen in Stented Patients) [74]. This registry was a prospective, multicenter, observational study conducted in the US and Europe between July 2009 and December 2010. The inclusion criteria were successful stent implantation in at least one native coronary artery and the patient was discharged on DAPT. The registry was designed to examine the impact of different modes of DAPT cessation on incidence of clinical adverse events.

The endpoint of the study during the 2-year follow-up was any DAPT cessation and/or the occurrence of any ischemic or bleeding adverse events. Coronary thrombotic events were defined as the occurrence of a stent thrombotic (ST) of a non-stent-related coronary thrombotic complication (spontaneous myocardial infarction). MB was defined as the occurrence of Bleeding Academic Research Consortium type 3 or 5 bleed [71]. Anemia was classified as a hemoglobin level <12 g/dL in men and <11 g/dL in women.

Previously selected independent predictors for both CTE and MB created multivariate risk models. Using the fully adjusted regression coefficients from each respective model, the integer risk scores for each outcome were generated. Integer Risk Score for MB consisted of the following covariates: age (years) (<50—0 points, 50–59—1 points, 60–69—2 points, 70–79—3 points, ≥80—4 points); BMI (kg/m^2^) (<25—2 points, 25–34.9—0 points, ≥35—2 points), current smoking (yes—2 points, no—0 points); anemia (present—3 points, absent— 0 points); CrCl < 60 mL/min (present—2 points, absent—0 points); triple therapy on discharge (yes—2 points, no—0 points). The range of integer scores for MB was 0 to 14, with patients categorized at low (0 to 3), intermediate (4 to 7), and high (≥8) bleeding risk.

Integer Risk Score for CTE events consisted of the following covariates: DM (none—0 points, non-insulin-dependent—1 points, insulin-dependent—3 points); ACS (no—0 points, yes, Troponin-negative—1 points, yes, Troponin-positive—2 points); current smoking (yes—1 points, no—0 points); CrCl < 60 mL/min (present—2 points, absent—0 points); prior PCI (yes—2 points, no—0 points); prior CABG (yes—2 points, no—0 points). For CTEs, the scores ranged from 0 to 10, and patients were grouped according to low (0 to 2), intermediate (3 or 4), and high (≥5) thrombotic risk. The absolute risk differences in CTE and MB for each patient could be treated as an indirect marker of a patient’s overall ischemic and bleeding risk. Differences greater than 0 indicate that risks from thrombosis exceed those of bleeding (and for these patients prolonged time of DAPT should be considered), whereas risk differences less than 0 indicate the opposite. The PARIS risk score has been thoroughly validated within the external validation cohort. An external validation was performed using data from ADAPT-DES (Assessment of Dual Antiplatelet Therapy With Drug-Eluting Stents) [75]. The final models of risk score exhibited discrimination ability: for the CTE model c statistic was 0.70 for the entire cohort and for MB model in the overall population a c statistic was 0.72. The final model of risk score exhibited moderate performance in the external validation cohort (the c statistic of 0.65 and 0.64 for CTE and MB risk scores, respectively).

#### 3.5.5. PRECISE DAPT Risk Score

The PRECISE DAPT score [76] is another system for assessing the risk of bleeding complications in patients undergoing DES implantation procedures (both in stable coronary disease and in ACS). This scale, apart from the assessment of the risk of bleeding within 1 year after discharge from the hospital, is also a valuable tool for the clinician, allowing them to assess the most optimal duration of DAPT at the start of therapy. DAPT (aspirin and P2Y12 inhibitors) significantly reduces the risk of ischemic recurrences in patients after coronary stent implantation. On the other hand, this benefit is counterbalanced by higher bleeding risk, which is linearly related to the treatment duration. Both the risk of ischemia and the risk of bleeding negatively affect the survival rate in this group of patients. As standard, according to the ESC guidelines, the duration of DAPT after stent implantation in ACS is 12 months. This time, depending on clinical indications, disease burden, and the patient’s individual risk profile, can be shortened or extended. On the one hand, shortening DAPT duration from 12 months to 6 or 3 months significantly reduced bleeding incidence; on the other hand, prolonged treatment beyond 12 months reduced the incidence of both stent-related and non-stent-related ischemic events.

The scale was based on the analysis of eight multicenter, contemporary, randomized clinical trials (RCT) (14,963 patients were enrolled in 139 different clinical sites from 12 countries worldwide) concerning patients treated with DAPT after coronary stenting. DAPT consisted of an association of aspirin plus a P2Y12 inhibitor, most commonly clopidogrel (88%). The primary endpoint of this analysis was out-of-hospital bleeding defined according to the TIMI definition [69] occurring 7 days or later after the initial invasive procedure. Using Cox proportional hazards regression, a multivariable model of risk assessment for bleeding was created. A final five-item bleeding risk score was developed from the previous model. Selected predictor values were scaled and rounded to a score with integer values between 0 and 100. The PRECISE DAPT scale includes age, CrCl, hemoglobin, white-blood-cell count at baseline, and previous spontaneous bleeding. The ability to identify patients at high bleeding risk was visualized by Kaplan-Meier cumulative bleeding incidence curves. Use the web calculator or mobile app to calculate the scale value and the corresponding bleeding rate. Kaplan-Meier bleeding rates were consistently separated by score quartiles (very low risk: ≤10; low risk: 11–17; moderate risk: 18–24; and high risk: ≥25). In five of the eight analyzed studies, patients were randomly assigned to two possible DAPT duration patterns: 12 or 24 months (5050 patients) and 3 or 6 months (5031 patients).

The effect of DAPT duration on the risk of a bleeding or ischemic episode across bleeding risk score quartiles was also analyzed. Significant increase in bleeding with a long (12–24 months) rather than short (3–6 months) duration of treatment was observed in patients at high bleeding risk, but not in those without a high bleeding risk profile (very low risk, low risk, and moderate risk). Concurrently, longer DAPT duration reduced the composite ischemic endpoint (MI, definite ST, stroke, target vessel revascularization) in those at non-high bleeding risk, but not in those at high bleeding risk. A scale value of 24 is indicated as the cutoff point; in patients who achieved ≥24 points, the risk of bleeding with a longer duration of DAPT (over 12 months) significantly exceeded the benefits of reducing the incidence of ischemic events in this group of patients. A similar relationship was observed in the group of patients with MI: PRECISE DAPT score ≥25 showed a significant increase in TIMI bleeding incidence after longer than 12 months DAPT duration, whereas those with a non-high PRECISE-DAPT risk score (<25) did not. At the same time, longer DAPT duration reduced the composite ischemic endpoint at a non-high PRECISE-DAPT score, but not in those with a PRECISE-DAPT score ≥25. The PRECISE-DAPT score showed a good discriminatory capacity: c statistic 0.73 (95% CI 0.61–0.85) for out-of-hospital TIMI major or minor bleeding and 0.71 (95% CI 0.57–0.85) for TIMI major bleeding within 12 months after stent implantation. The score discrimination was consistent regardless of the clinical subgroups of patients (stable coronary artery disease vs. MI) or treatment with clopidogrel vs. ticagrelor. The PRECISE-DAPT score has been thoroughly validated in the context of two independent PCI-treated populations. An external validation was performed using data from the Platelet Inhibition and Patient Outcomes (PLATO) registry (8595 patients) [77] and from the BernPCI registry (6172 patients). The PLATO trial included patients with STEMI or NSTEMI randomly assigned to receive DAPT with either clopidogrel or ticagrelor in addition to aspirin for up to 12 months. The BernPCI registry included all patients undergoing PCI at Bern University Hospital, Switzerland, between February 2009 and December 2014. The c-indices for TIMI major or minor bleeding were 0.70 (95% CI 0.65–0,74) in the PLATO trial and 0.66 (95% CI 0.61–0.71) in the BernPCI registry.

#### 3.5.6. DAPT Risk Score

The DAPT risk score [78] is one of several scales to assess the risk of post-discharge bleeding in patients after stent implantation and treatment with DAPT. The purpose of using the DAPT risk score in patients after coronary stent implantation was an attempt to identify patients for whom the expected benefits related to the reduction of ischemic events resulting from DAPT prolongation beyond 12 months would outweigh the increased risk of bleeding. The DAPT score is based on a secondary analysis of the DAPT study [79], which was conducted from August 2009 to May 2014 in 11 countries and enrolled 25,682 patients after PCI with DES or BMS treated with thienopyridine plus aspirin for 12 months. At 12 months, eligible patients who were free from major bleeding and ischemic events were randomized to continued thienopyridine + aspirin therapy (5862 patients) and aspirin + placebo (5786 patients) for the next 18 months. The primary ischemic endpoint was a composite of MI or Academic Research Consortium definite or probable ST [80] and the primary bleeding endpoint was moderate or severe bleeding, as defined by the GUSTO criteria [70]. It is assumed that prolongation of DAPT reduces the risk of ischemia at the expense of increasing the risk of bleeding. When creating the DAPT score it was assumed, however, that some heterogeneity in the effect of DAPT prolongation is possible; namely, in some patients, prolongation of DAPT will reduce the risk of ischemia without significantly increasing the risk of bleeding.

In order to create a model to identify these patients, separate scales were created using Cox regression methods for the risk of ischemia and the risk of bleeding. For each patient after randomization, a “benefit-risk difference” was determined, the value of which was the absolute difference between the predicted ischemic reduction and predicted bleeding increase (resulting from randomizing patients to the group treated with thienopyridine plus aspirin). A linear regression model was created, using benefit-risk difference as the outcome and all predictors from the ischemia and bleeding models. Variables that contributed more than 1% of the observed variation in estimated benefit-risk difference were included in a final clinical score. All variables were assigned an integer score of 1 or 2 (or −1 to −2) based on the beta coefficient. The range of scores was between −2 and 10, assigned points as follows: 0 for age <65, −1 for age 65–<75, −2 for age ≥75, 2 for vein graft PCI, 1 for current cigarette smoker or within past year, 1 for DM, 1 for MI at presentation, 1 for stent diameter <3mm, 2 for history of CHF or LVEF <30%, 1 for prior PCI or prior MI, and 1 for paclitaxel-eluting stent. In DAPT study derivation cohort higher score quartile was associated with higher rates of ischemia events, whereas lower score quartiles were associated with higher rates of bleeding events. Furthermore, patients randomized to aspirin + thienopyridine therapy who were assigned to higher score quartiles presented larger observed risk reductions in incidence of ischemic event, patients assigned to lower score quartiles presented greater observed risk increases in bleeding. Its median was considered to be the cut-off value of the scale. In the group of patients with predictive scores ≥2 (*n* = 5917), randomization to continued thienopyridine was associated with larger reductions in risk of ischemia compared with those with scores <2, (*n* = 5731). Conversely, randomization to aspirin + thienopyridine was associated with smaller increases in bleeding among high score patients compared with low score patients. The DAPT risk score was externally validated within the PROTECT Trial [81] which enrolled patients undergoing PCI and randomized to receive sirolimus-eluting (SES) vs. zotarolimus-eluting stents (ZES). The study was conducted from June 2007 to July 2014 in 36 countries. Patients without an ischemic or hemorrhagic event within the first 12 months of observation were enrolled in the validation cohort (*n* = 1836). Both the primary risk models (ischemic and bleeding risks) and the final prediction score performance in stratifying risks of ischemic and bleeding events were validated. In PROTECT Trial patients were not randomized to different durations of DAPT (DAPT duration was modified by treatment indication). The models used to derive the predictive score showed modest discrimination ability in the validation cohort (ischemic model c statistic 0.64, 95% CI 0.58–0.70, bleeding model c statistic 0.64, 95% CI 0.55–0.73). The ability of the clinical prediction score to stratify ischemic and bleeding risk was evaluated by comparing overall rates of ischemic and bleeding events among patients with high vs. low score in the validation cohort. The rate of ST or MI between 12–30 months after PCI was higher among high score patients compared with low score patients (1.5% vs. 0.7%, respectively). Rates of moderate or severe bleeding were not significantly different by score group (0.36% among high score patients and 0.52% among low score patients).

## 4. Discussion

The utility of modern risk assessment systems in patients treated for MI is indisputable. This is confirmed both by the official guidelines issued by the ESC and by daily clinical practice. The benchmark risk score should be simple to use, accessible at the bedside, and easy to interpret. Risk assessment complements the clinical assessment of the patient and should be repeated in a different moment in the timeline of ACS (from admission, through treatment, discharge from hospital, to long-term post-hospital care). The risk assessment scores are valuable tools for the physician, supporting the process of making clinical decisions, pointing to the optimal pharmacotherapy, helping to determine the duration of hospitalization, and facilitating the selection of post-hospital management strategies. When analyzing the increasing number of emerging risk assessment systems, several groups can be distinguished according to the following criteria:

1. assessed endpoint: risk of ischemia/death or bleeding risk or risk of a composite endpoint (death and MACE),
2. time perspective of the risk assessment: short-term assessment of the risk (in the in-hospital period or within 30 days) or long-term assessment of risk (different duration of the follow-up period),
3. spectrum of analyzed ASCs: assessment of the risk in patients with STEMI or NSTEMI/UA or patients with the full spectrum of ACS,
4. the moment at which the risk analysis is performed: assessment based on the basic clinical and laboratory parameters collected on admission to the hospital or risk assessment based on the analysis of other data obtained during hospitalization or performed at the end of hospitalization,
5. how to use the scale: simple, bedside nomograms or electronic calculators (via internet or mobile app).

In the course of daily clinical practice, the physician must select the appropriate risk scale for the appropriate patient at the appropriate time point of treatment. The area of application of individual risk scales often overlaps, which makes it even more difficult to choose the appropriate scale. When using the risk score, it is worth being aware of the size and clinical characteristics of the cohort on the basis of which the given risk assessment system was created, the criteria that exclude patients with derivation cohort, the methods of its treatment (conservative vs. invasive), the selection of pharmacotherapy (including the use of new antiplatelet drugs), and clinical presentation (STEMI vs. NSTEMI/UA). The closer the characteristics of the research cohort to the clinical scenario we are assessing, the more the predictive value of the scale increases. Proper, extensive external validation of the scale is also very important. Due to the rapid development of interventional cardiology methods and devices in recent years, it is also worth paying attention to the period in which patients were enrolled in the derivation cohort. It is impossible to create one perfect scale applicable in every situation and in every clinical scenario; however, in everyday practice it seems impractical to use separate scales for each of the possible risks. The physician using a given risk assessment system should know its limitations as well as its strengths. The first of the objections concerning many scales created on the basis of the clinical randomized trials are the exclusion criteria used when recruiting for the trial. As a result, the research cohort does not reflect the real population of patients. A brief description of the potential advantages and limitations of each risk score is presented below.

The TIMI scale is recommended by the ESC to assess the risk of death within 30 days of a STEMI patients. The risk assessment is made on admission to the hospital. Despite the undoubted advantages (numerical derivation cohort, simple scale, easy to apply at the bedside, does not require electronic devices, validation on a large group of patients), it should be remembered that the scale was created over 20 years ago and was based on the analysis of patients with STEMI treated exclusively by fibrinolysis. Nowadays, the vast majority of patients are treated only invasively and the introduction of new antiplatelet drugs and stents significantly changes the fate of patients. Nevertheless, a paper was published in 2014 [82] on the use of the TIMI score in patients treated with PCI (the derivative cohort consisted of 8073 PCI-treated STEMI patients, enrolled to the prospective, observational Belgian STEMI registry from January 2007 to February 2011). TIMI risk score still provides acceptable discrimination for the prediction of 1-year mortality (c statistic 0.72). The version of the TIMI scale dedicated to patients with NSTEMI/UA (currently not recommended for use by the ESC guidelines) should also be mentioned here. Like the previous one, this is the scale used on admission to hospital and is designed to assess the risk of death, heart attack, or urgent revascularization within 14 days of admission to hospital. In this case, as before, the main limitation of the scale is the derivation cohort maladjustment (patients enrolled in clinical trials in 1996–1998, not treated invasively), as well as the moderate discriminating value of the scale (c statistic 0.65). The last scale from the TIMI group of scales is the TIMI DYNAMIC risk score—a simple-to-use clinical scale that allows one to assess the risk of death of a patient with STEMI within 1 year of discharge. This scale is used to reclassify patients with STEMI based on in-hospital events. It is worth noting that the DYNAMIC TIMI scale is one of the few that takes into account the occurrence of in-hospital events (recurrent MI, stroke, major bleed, arrythmia, renal failure, CHF, or cardiogenic shock) in the long-term risk assessment process. One should also be aware that the ExTRACT TIMI 25 study had quite numerous exclusion criteria (e.g., cardiogenic shock, renal failure) and was based on fibrinolytic therapy. The scale was validated externally on a small cohort of patients (1829) enrolled in the TRITON TIMI study, where 99% of patients underwent PCI and were treated with prasugrel/clopidogrel. Despite differences in the characteristics of the studied cohorts, predictive capacity of TIMI Dynamic risk score remained consistent for 1-year mortality with c statistic of 0.81.

The GRACE risk score is currently the most widely used and best validated scale for assessing the risk of MI patients. ESC recommends it as the basic scale for assessing the risk of patients with NSTEMI (class II a LOE B). It should also be mentioned that the GRACE scale was included in the decision-making process regarding the treatment of patients with NSTEMI. The GRACE score above 140 points qualifies the patient to the group of high-risk patients, which is related to the recommendations for implementing an early routine invasive strategy within 24 h of admission (class I LOE A). Over the last 20 years, new versions of the GRACE scale have been developed, based on subsequent analyses of patients from the GRACE registry. The advantages of this scale include the simplicity of use that allows it to be used at the patient’s bedside. Initially, the score result was read on a special nomogram, and an internet calculator/mobile phone application was developed for subsequent versions of the scale. The scale was created on the basis of a very large international registry of patients with the entire spectrum of ACS, which favors a faithful reflection of the real population of patients with MI (the scale can be used both when assessing the risk of patients with STEMI and NSTEMI). The subsequent versions of the scale were based on new research cohorts separated in the following years from the GRACE register, which meant that the new versions of the scale took into account the progress in the development of pharmacotherapy and invasive treating of MI. The subsequent revisions of the GRACE scale give the physician a unique opportunity to assess both short-term and long-term risk; risk assessment of death as well as risk assessment of the composite endpoint of death or recurrence of non-fatal MI. Risk assessment using the GRACE scale can be performed both on admission (assessing in-hospital risk) and on discharge from the hospital (assessing the risk over a period of 6 months, 1 year, and 3 years). The scale also introduced the possibility of an alternative replacement of the serum creatinine concentration value with information about the patient’s renal failure (in the absence of data) and the value of the Killip class with information about the use of diuretics by the patient previously. This allows for wider, even easier, and more common use of the GRACE scale in assessing the risk of patients with MI. The subsequent versions of the GRACE scale have a very good predictive value, assessed both in the research cohort and in the validation cohorts. The most serious allegations against the GRACE scale include (despite subsequent updates) the maladjustment of the research cohort to the modern population (GRACE 2.0 was based on patients enrolled in the registry between January 2002 and December 2007). Doubts are also raised by the small size of the study cohort (1274 patients), on the basis of which a model for the analysis of the risk of death within 3 years from admission to the hospital was developed.

Another risk assessment system is the ACTION Registry-GWTG risk model [43]. In principle, it is a simple scale based on clinical, electrocardiographic, and laboratory parameters collected on admission to hospital, used to assess the in-hospital risk of patients with both STEMI and NSTEMI undergoing modern invasive treatment. Its advantages include ease of use, a very large derivative cohort, good discriminating value (c statistic 0.83), and use in the entire spectrum of currently treated ACS. The limitations of the scale include: the scale was based on the voluntary registry, patients transferred from one hospital participating in the study to another present a question of outcome attribution, the possibility of assessing the risk of death only during hospitalization, and no external validation.

EPICOR risk calculator [45] assesses the risk of death within 2 years of hospital discharge following MI. Its advantages include the possibility of risk assessment in patients with the entire spectrum of ACS, a very numerous, heterogeneous derivation cohort from Europe, Latin America, and Asia, and high predictive value (c statistics 0.8). The disadvantages include undoubtedly the complexity of the scale (17 parameters), the need to assess subjective factors (EuroQoL—quality of life generic questionnaire), the fact that some in-hospital complications (bleeding, stroke, infection) were not included in the model, there was no external validation of the risk model, and it is not possible to assess the risk of developing other endpoints than death (e.g., non-fatal ischemic events).

The ACEF scale is another system for assessing the risk of patients after an MI [56]. The advantages include the simplicity of the scale, the ability to assess the risk of patients with the entire ACS spectrum, the ability to assess both short-term (30 days) and long-term (1 year) risk, the derivation cohort was treated with modern methods of invasive cardiology, and the ACEF score was a predictor of not only all-cause mortality, but also of MACCE and transient ischemic attack/stroke. The limitations of this scale include a small research cohort and the lack of external validation.

The CRUSADE [62] scale is the first of the scales dedicated to risk of bleeding; this risk score is officially recommended by the ESC in the guidelines for the treatment of NSTEMI (class II b, LOE: B). The scale is based on eight simple clinical parameters collected on admission to hospital and assesses the risk of major bleeding during hospitalization. The advantages of this system include a very numerous derivation cohort, a relatively easy way to use it in everyday practice (it does not require the use of electronic devices), a varied treatment of patients from the research cohort (invasive, conservative, and cardiosurgical treatment), and the scale is based on the CrCl assessment, not serum creatinine concentration (CrCl better reflects kidney function). On the disadvantage side, it should be noted that the study took place in the years 2003–2006, the study cohort included patients with only NSTEMI (patients with UA were excluded), the study cohort also excluded patients taking warfarin, transferred to other hospitals, patients who died within 48 h of admission, the analysis of bleeding in patients undergoing CAGB was limited only to the preoperative period, only hematocrit levels (not hemoglobin) were analyzed, the scale does not include information about history of prior bleeding or bleeding diathesis, patients enrolled in cohort study were not treated with new antiplatelet drugs, and. The discriminatory capacity of CRUSADE score was moderate (c statistic 0.72).

The ACUITY risk score [64] is another of the scales recommended in the ESC guidelines for the treatment of NSTEMI. The scale is based on seven clinical parameters collected on admission to hospital and assesses the risk of major bleeding within 30 days of hospital admission (it was also adapted to the nine-component version to assess mortality 12 months after discharge from the hospital). The advantages of this system include a relatively easy method of application in everyday practice (it does not require the use of electronic devices), a varied treatment of patients from the study cohort (invasive, conservative and cardiosurgical treatment), the possibility of assessing short- and long-term risk, and the study cohort was composed of patients with both NSTEMI/UA and STEMI. The limitations of this scale include the currently rare anticoagulant treatment during PCI used in the study cohort (administration of, inter alia, bivalirudin plus and GPI or bivalirudin in monotherapy), patients were not treated with new antiplatelet drugs (clopidogrel was used, ticlopidine in case of allergy to the latter), no external validation of the scale, and the moderate discriminant value of the scale (c statistic 0.74). Both of the above-mentioned risk scales (CRUSADE and ACUITY) do not take into account changes in the practice of interventional management in the form of a very frequently used radial approach, which is a proven factor reducing the number of bleeding complications [83].

BleeMACS risk score [67] is a clinical tool for risk estimation of 1-year post-discharge serious bleeding in patients with MI. The advantages of this risk score include the possibility of risk assessment in patients with the entire spectrum of ACS (the study cohort included patients with all forms of ACS); a very numerous, heterogeneous study cohort from North and South America, Europe, and Asia, which guarantees a true representation of the population of patients with MI; a seven-factor simple risk score model; patients enrolled in study and validation cohort were partially treated with new antiplatelet drugs; and the scale was validated both internally and externally on a large group of patients. The value of the c statistic for the scale in the research cohort was 0.71, which is a moderate value. It is noteworthy that, unlike the risk scores discussed above, BleeMACS has been positively validated for use in patients receiving oral anticoagulation. The disadvantages of this risk assessment system include the need to use an electronic calculator (application on a mobile device) to determine the final value of cumulative incidence of bleeding during 1 year of observation, as well as not taking into account potential changes in antithrombotic therapy, such as switches between antiplatelet drugs or DAPT discontinuation. The antithrombotic treatment regimen was intentionally omitted during construction of the risk score because in daily clinical practice the prescription of antithrombotic drugs conditioned by the individual risk of bleeding. However, an analysis examining the performance of the BleeMACs score in different antithrombotic regimens was assessed in the SWEDEHEART population. The score discrimination capacity was similar in the different DAPT regimens (c statistic 0.65 for aspirin plus clopidogrel and for aspirin plus ticagrelor, and of 0.63 for aspirin plus prasugrel; c statistic 0.69 for oral anticoagulation plus a single antiplatelet drug and of 0.60 for triple therapy-oral anticoagulation plus DAPT) as well as in population non-PCI patient.

The PARIS risk score [73] is actually a composite of two separate scales and is an interesting example of a system that includes the ability to assess the overall major risks of a patient with MI. The scale assessed the risk of bleeding and thrombotic events within 2 years of PCI with DES stent implantation. The advantages of this system include possibilities of assessing the balance between the risk of ischemia and the risk of bleeding in a given patient, which may be helpful in determining the duration of dual antiplatelet therapy (the possibility of extending beyond the standard period). The authors of the study suggested that, in the case of CTE ≥5, extension of DAPT duration (regardless of the MB level) should be considered. This solution requires further research. The research cohort was based on a large, international observational study conducted in 2009–2010 and patients treated with oral anticoagulants were not excluded from the cohort, all of which contributes to a good reflection of the actual patient population. The scale is based on simple clinical and laboratory parameters assessed during discharge from the hospital (it can be used at the patient’s bedside). The Paris scale for long-term risk assessment focused on adverse events mainly related to the duration of DAPT (ST and bleedings), rightly not paying much attention to PCI-related periprocedural bleeding (which has a greater impact on short-term in-hospital mortality). The undoubted disadvantages of this risk assessment system include the fact that the vast majority of the study cohort consisted of patients with stable angina undergoing elective PCI (ACS constituted only 37.8% of all patients). Another aspect limiting the use of the PARIS scale is the fact that patients were mainly treated with clopidogrel, which does not reflect the current trends in pharmacotherapy related to PCI. It is also worth mentioning the moderate discriminating capacity of the scale in the external validation cohort (the c statistic of 0.65 and 0.64 for the thrombotic and bleeding risk scores, respectively).

The DAPT risk score [78] is used to assess the risk of post-discharge bleeding patients after stent implantations and treated with DAPT. Assessment with this scale should take place after 12 months of DAPT. The derivation cohort consisted of patients enrolled in DAPT—a large international clinical trial conducted in 2009–2014 [79]. Unfortunately, only 73.8% of these patients were ACS patients. It is a scale that is relatively simple to use, containing both clinical parameters related to PCI as well as echocardiography. The disadvantages of this scale include the fact that the study cohort excluded patients undergoing long-term anticoagulant therapy, patients with elective surgery requiring discontinuation of antiplatelet therapy for >14 days, and patients with a life expectancy <3 years (due to inclusion criteria for the DAPT study). Most of the patients enrolled in the DAPT study received treatment with clopidogrel + aspirin, which differs from modern standards. Some patients had an implanted BMS (14.4%), and most of the implanted DES were of the first generation (which may overestimate the frequency of ST/ischemic events). The ischemic model and bleeding model showed similar moderate discrimination (c statistic 0.70, 0.68; respectively). It is also worth noting that the statistical test for interaction did not show a difference in the effect of continuation of long-term DAPT on mortality in high vs. low prediction score groups. Both the primary risk models (ischemic and bleeding risks) and the final prediction score performance in stratifying risks of ischemic and bleeding events were validated. The models used to derive the predictive score showed modest discrimination ability in the validation cohort (ischemic model c statistic 0.64, bleeding model c statistic 0.64) [81]. The final risk score was validated on a limited basis, with a retrospective analysis in 1970 patients and calculation of the score at a different time point (6 vs. 12 months) than in the study cohort used to develop the scale [84]. The latest ESC guidelines recommend a 12-month duration of DAPT in patients after both STEMI and NSTEMI (class I, LOE: A) [7,8]. This period may be changed in the presence of important contraindications. The ESC guidelines for the treatment of NSTEMI recommend the use of risk scores designed to assess the benefits and risks associated with different durations of DAPT (class IIb, LOE: A). The two risk assessment systems described above (DAPT and PARIS) have the same goal—to assess the possibility of extending the DAPT beyond 2 years. Both these systems complement each other. It is worth noting that one of these systems was developed on the basis of a clinical trial (DAPT), and the other was based on an observational register (PARIS), which has certain advantages and limitations related to it. For both scales, patients in the study cohort were treated mainly with clopidogrel. The PARIS score should be done after PCI and the DAPT score should be done after 12 months of DAPT. The latest ESC guidelines for the treatment of NSTEMI quite thoroughly discuss the issue of prolonging the duration of DAPT beyond 12 months, suggesting a combination of ticagrelor + aspirin first, and then prasugrel + aspirin or clopidogrel + aspirin if the patient is not eligible for ticagrelor treatment. This treatment regimen should be used in patients at high risk of ischemic events and without increased risk of major bleeding (class IIa LOE: A) and in patients at moderate risk of ischemic events and without increased risk of major bleeding (class IIb LOE: A). The guidelines accurately provide a definition of high and moderate risk of ischemic events as well as a definition of an increased risk of major bleeding. In the case of guidelines for the treatment of STEMI infarction, administration of DAPT >12 months (ticagrelor 60 mg twice a day) may be considered in patients at high risk of ischemic events who tolerated the current DAPT without bleeding complications (class IIb LOE: B). As can be seen, the ESC guidelines do not provide for the use of the PARIS or DAPT scale to assess the possibility of extending the duration of DAPT above 12 months.

The PRECISE DAPT [76] score is another system for assessing the risk of bleeding complications in patients undergoing PCI with DES stent implantation and receiving DAPT. The scale assesses the risk of a bleeding event within 1 year of surgery in both patients with stable angina and MI. One of the advantages of this score is undoubtedly its simplicity: it consists of five parameters (two clinical and three laboratory) and should be used as soon as the patient is admitted to the hospital. It is true that, in order to determine the predicted incidence of bleeding within 1 year of DAPT, you need to use an internet calculator/mobile app; however, at the patient’s bedside, a physician can classify the patient as one of the bleeding risk groups. The score is recommended by the ESC in the NSTEMI treatment guidelines as a tool for assessing the possibility of reducing DAPT time (in patients with a score ≥25, discontinuation of P2Y12 inhibitor treatment up to 3 mc may be considered) (class IIa, LOE: B). In patients at high risk of bleeding on the PRECISE-DAPT score (i.e., ≥25 points), prolonged DAPT was associated with no benefit for ischemic events with a significant increase in the risk of bleeding complications. On the other hand, longer therapy in patients not at high risk (i.e., with PRECISE-DAPT scores <25) does not increase the risk of a bleeding events and is associated with a significant reduction in the incidence of ischemic events. The advantages of this scale also include a very large study cohort based on participants of eight multicenter, international, modern, RCTs, which well reflects the actual population of patients treated with DAPT. Patients with MI (the entire ACS spectrum) accounted for 55.6%. Unfortunately, randomized trials have their limitations, one of which was the exclusion of patients taking oral anticoagulants from the study cohort. The PRECISE-DAPT score showed a moderate discriminatory capacity: c statistic 0.73 for out-of-hospital TIMI major or minor bleeding and 0.71 for TIMI major bleeding. Another limitation of the scale is the fact that the majority of patients in the study cohort were treated with the aspirin plus clopidogrel (88%). The score has undergone extensive external validation in the context of two independent PCI-treated populations (patients with stable coronary disease and ACS). The PRECISE DAPT score showed an average discrimination ability for TIMI major or minor bleeding (c statistics ranging from 0.66 to 0.7 depending on the validation cohort). ACS patients from validation cohorts have been treated with all three P2Y12 inhibitors: clopidogrel, prasugrel, and ticagrelor. When only patients with ACS who underwent PCI and were treated with prasugrel or ticagrelor (4424) were included in the validation cohort, the scale showed little predictive value for major bleeding over a mean follow-up of 14 months (statistic c 0.653). Due to the fact that the PRECISE DAPT scale has not been prospectively assessed in RCT, its value in terms of improving patient outcomes is still unclear.

## 5. Conclusions

Over the past decade, a wide variety of risk assessment systems have been developed for patients with myocardial infarction. In the article, the authors described only the more important scales, because when analyzing the literature, one can find many reports on new risk scales, which are beyond the scope of this article. Despite the multitude of these scales, none of them seems to be perfect. The question is what criteria should be met by risk assessment systems in the future and how they should be built. It seems obvious that the discriminating value of the scale for indicating patients at high risk of death will depend on the size and diversity of the study cohort on the basis of which the score will be created. The larger and more diverse the study cohort, the greater the chance of a more complete reflection of the real population of patients with MI. Enrolling patients from different countries/continents in the study cohort will allow for a significant diversity of the population, as well as taking into account the differences in the organization of local health systems. Risk assessments systems are based either on large international randomized trials or on observational ACS registries. Both have advantages and disadvantages as described above in the consideration of the practical limitations of the risk scores. It has been confirmed that the relationship of dependencies between individual risk factors and outcomes may be less apparent in randomized as opposed to observational studies [85]. The perfect solution seems to be to create a study cohort based on the largest possible observational ACS registry and then validate the developed risk score in prospective RCT. When analyzing individual risk assessment systems, it can be concluded that there is a certain group of parameters (clinical, laboratory, procedural, etc.) that should always be taken into account when constructing any risk score for a patient with MI. The predictive value of the same parameter may differ depending on the profile of the studied population, the used invasive and pharmacological treatment methods, as well as change its value within the same population depending on the time frames adopted during the analysis (short-term assessment vs. long-term assessment). Therefore, the continuous development of cardiology will force the creation of new risk assessment scores, which must be constantly updated with changes in the characteristics of the population of patients with MI, treatment methods, and current pharmacotherapy. The wide application of modern medical technologies has resulted in the emergence of new, previously unknown, unintended complications related to the applied pharmacological and surgical treatment—the so-called serious adverse events (SAE), which go far beyond the definition of MACCE or definitions of major bleeding. SAE are defined according to the Harvard Medical Practice Study definition: “an unintended injury or complication that results in disability at the time of discharge, death or prolonged hospital stay caused by health care management rather than by the patient’s underlying disease process” [86]. An example of SAE may be complications related to PCI (coronary artery dissection, aortic dissection, coronary artery rupture, contrast-induced nephropathy) as well as related to intensive medical therapy, which is increasingly required in patients with MI (pneumothorax, infectious complications). Although SAEs are not always associated with patient death, they significantly affect the duration of hospitalization and long-term prognosis. Another issue that is not discussed more broadly in the context of risk assessment of a patient with MI is the risk assessment resulting from the overall logistics of the treatment process. It is not about time delays during the infarction treatment procedure, the effect of which on the effectiveness of treatment is well described [87], but about improper organization of the workplace, the impact of the time of day or night, day of the week (weekday, weekend, holiday day) in which the patient is admitted to the hospital (or undergoing invasive procedure), or finally due to the experience of the staff or their workload on a given day. In the literature, there are only individual reports on the importance of these issues for the final results of treatment in a patient with MI [88]. Additionally worth mentioning is the phenomenon of “treatment risk paradox” consisting in the unintentional less aggressive treatment of high-risk patients. It has been proven that as the GRACE score increases, less aggressive treatment is used (e.g., patients undergo coronary angiography less frequently) [89]. The reasons for this may be presumed to be in the physician’s reliance only on his own clinical assessment when assessing the patient with MI. Patients at high risk of dying from MI are elderly patients, with more comorbidities, and with more advanced CAD (multivessel disease), which undoubtedly translates into a significant increase in the risk of SAE during invasive procedures and follow-up. This may to some extent explain the treatment risk paradox. It should be remembered that the currently recommended risk scores in MI patients assess the risk only of classic ischemic or bleeding events. Currently, there are no risk scores assessing the risk of other adverse events associated with the invasive treatment than MACCE. Only the assessment of the balance between the risk of a traditional ischemic/bleeding events (and the associated increased risk of death) and the risk of a SAE during invasive treatment, and the translation of this balance into the final results regarding patient mortality, would allow clinicians to identify the group of patients with MI that will benefit from the implemented invasive treatment. Looking to the future, the need to use risk assessment systems in the management of patients with MI appears to be unquestionable. Modern medicine, and above all cardiology, cannot exist without integral risk assessment systems. On the one hand, there are treatment standards set by the ESC guidelines (the guidelines are mainly based on the results of RCT) and, on the other hand, the assessment of their practical implementation in the form of mortality rates. The link between both sides are risk assessment systems. The future of risk assessment systems seems to be multi-directional. Firstly, the tendency is to create a comprehensive scale that takes into account all classic risks, as well as new types of risks related to the logistics of the treatment process. The second direction is the creation of risk scales assessing the risk of SAE, clinical scales assessing the interrelationships between logistic factors and the incidence of SAE, as well as scales that take into account both of the above-mentioned elements in the context of assessing short- and long-term prognosis. Another direction seems to be a return to the evaluation of parameters related to the PCI procedure; in recent years, there have been no scales developed that take into account the parameters of invasive procedures (the recently published scales taking into account these parameters were the ZWOLEE and SYNTAX scales published at the beginning of the 21st century). A solution worth attention also seems to be the creation of a scale combining both classic clinical parameters and hemodynamic parameters related to PCI, based on the population of patients treated with new antiplatelet drugs and with the use of new generation of DES.

## Data Availability

Not applicable.
